# PERK signaling pathway in bone metabolism: Friend or foe?

**DOI:** 10.1111/cpr.13011

**Published:** 2021-02-21

**Authors:** Jiachao Guo, Ranyue Ren, Kai Sun, Jinpeng He, Jingfan Shao

**Affiliations:** ^1^ Department of Pediatric Surgery Tongji Hospital Tongji Medical College Huazhong University of Science and Technology Wuhan China; ^2^ Department of Orthopedics Tongji Hospital Tongji Medical College Huazhong University of Science and Technology Wuhan China

## Abstract

Osteoblasts and osteoclasts participate in the process of bone remodelling to meet the needs of normal growth and development or repair pathological damage. Endoplasmic reticulum stress (ER stress) can break the intracellular homeostasis of osteoclasts and osteoblasts, which is closely related to abnormal bone remodelling. The double‐stranded RNA‐dependent protein kinase (PKR)‐like ER kinase (PERK) is a key transmembrane protein that regulates ER stress, and growing evidence suggests that the PERK pathway plays a crucial role in regulating bone metabolism under both physiological and pathological conditions. Based on the current findings, we summarized the main mechanisms involved in bone metabolism downstream of the PERK pathway, among which elF2α, FOXO1, CaN, Nrf2 and DAG play a role in regulating the differentiation of osteoblasts and osteoclasts. Importantly, strategies by the regulation of PERK pathway exert beneficial effects in preclinical trials of several bone‐related diseases. Given the importance and novelty of PERK pathway, we provide an overview and discuss the roles of PERK pathway in regulating bone metabolism and its impact on bone‐related diseases. We hope that the development of research in this field will bring a bright future for the treatment of bone‐related diseases.

## Graphical abstract

Effects of downstream changes caused by activation of PERK on osteoblastogenesis and osteoclastogenesis. Phosphorylated elF2a, Nrf2, DAG, FOXO1 and enhanced activity of CaN have different impacts on osteogenic and osteoclast differentiation, respectively. Green squares indicate the expression of the genes.

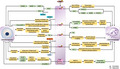

## 1 INTRODUCTION

Bone is a dynamic tissue that remodels constantly to prevent the accumulation of bone damage and at the same time maintain the mechanical strength of bone.^1^ The adult skeleton is renewed by remodelling every 10 years. It is estimated that 3‐4 million bone remodelling units (BRUs) are initiated every year, and 1 million BRUs are actively involved in bone turnover at any time.^2^ According to the definition, bone remodelling is a process where osteoclasts and osteoblasts work sequentially in the same BRU,^3^ and it is generally considered to be composed of four consecutive phases^4^: the activation phase when osteoclast progenitors are recruited to the surface of damaged bone; resorption phase when mature osteoclasts resorb the damaged bone; reversal phase when osteoclasts die and osteoblast progenitors are recruited; formation phase when mature osteoblasts produce new bone matrix.^5^ Osteoblasts are differentiated from bone marrow mesenchymal stem cells (BMSCs), which have multi‐directional differentiation potential: osteogenesis, chondrogenesis and adipogenesis.^6^ Mature osteoblasts secrete phosphatase, cooperate with calcium ions to form hydroxyapatite and calcify the bone matrix, eventually wrapped by matrix and degenerated into osteocytes.^7^ In addition to osteogenic function, osteoblasts can sense changes in intracellular homeostasis and regulate the function of neighbouring cells through autocrine and paracrine.^8^, ^9^ Bone marrow‐derived macrophages (BMMs) can differentiated into osteoclasts, which are multinucleated giant cells with bone resorption function. The process of osteoclast differentiation is regulated by two key cytokines: macrophage‐colony stimulating factor (M‐CSF) and receptor activator of nuclear factor κB ligand (RANKL).^10^ M‐CSF, which is mainly secreted by osteoblasts and bone marrow stromal cells, not only maintains the proliferation and survival of osteoclast precursors, but also have an important activation effect on the continuous expression of receptor activator of nuclear factor κB (RANK).^11^ RANKL is secreted by various cells such as osteoblasts, lymphocytes, bone marrow stromal cells and osteocytes, and promotes the differentiation and maturation of osteoclasts by activating RANK.^12^, ^13^, ^14^

The endoplasmic reticulum (ER) is a membrane organelle responsible for protein synthesis, modification, quality control and storage of calcium ions.^15^ Various pathological stimuli such as calcium ion imbalance, nutritional deprivation, oxidative stress or energy disturbance may break the intracellular homeostasis, too much unfolded or misfolded proteins are accumulated, and eventually induce the ER stress.^16^ Studies have found that ER stress is related to metabolic diseases, neurodegenerative diseases, immune deficiency‐related diseases and inflammatory reactions.^17^, ^18^, ^19^, ^20^ In order to restore the ER homeostasis and normalize its function, cells are equipped with evolutionarily conserved unfolded protein response (UPR).^21^ UPR can inhibit protein synthesis, promote the degradation of unfolded proteins, and has the ability to relieve ER stress and restore ER function.^22^, ^23^ UPR is mediated by three key transmembrane proteins: activating transcription factor 6 (ATF6), inositol‐requiring enzyme 1 (IRE1) and double‐stranded RNA‐dependent protein kinase (PKR)‐like ER kinase (PERK), these crucial molecular proteins participating in UPR have been the focus of disease mechanism research.^24^ In the skeletal system, UPR is an important regulator of bone metabolism.^25^ The focus of this review is on the molecular mechanism of the core elements of the PERK signalling in regulating bone metabolism and their effects on related bone diseases.

## 2 OUTLINE OF PERK SIGNALLING

**FIGURE 1:**
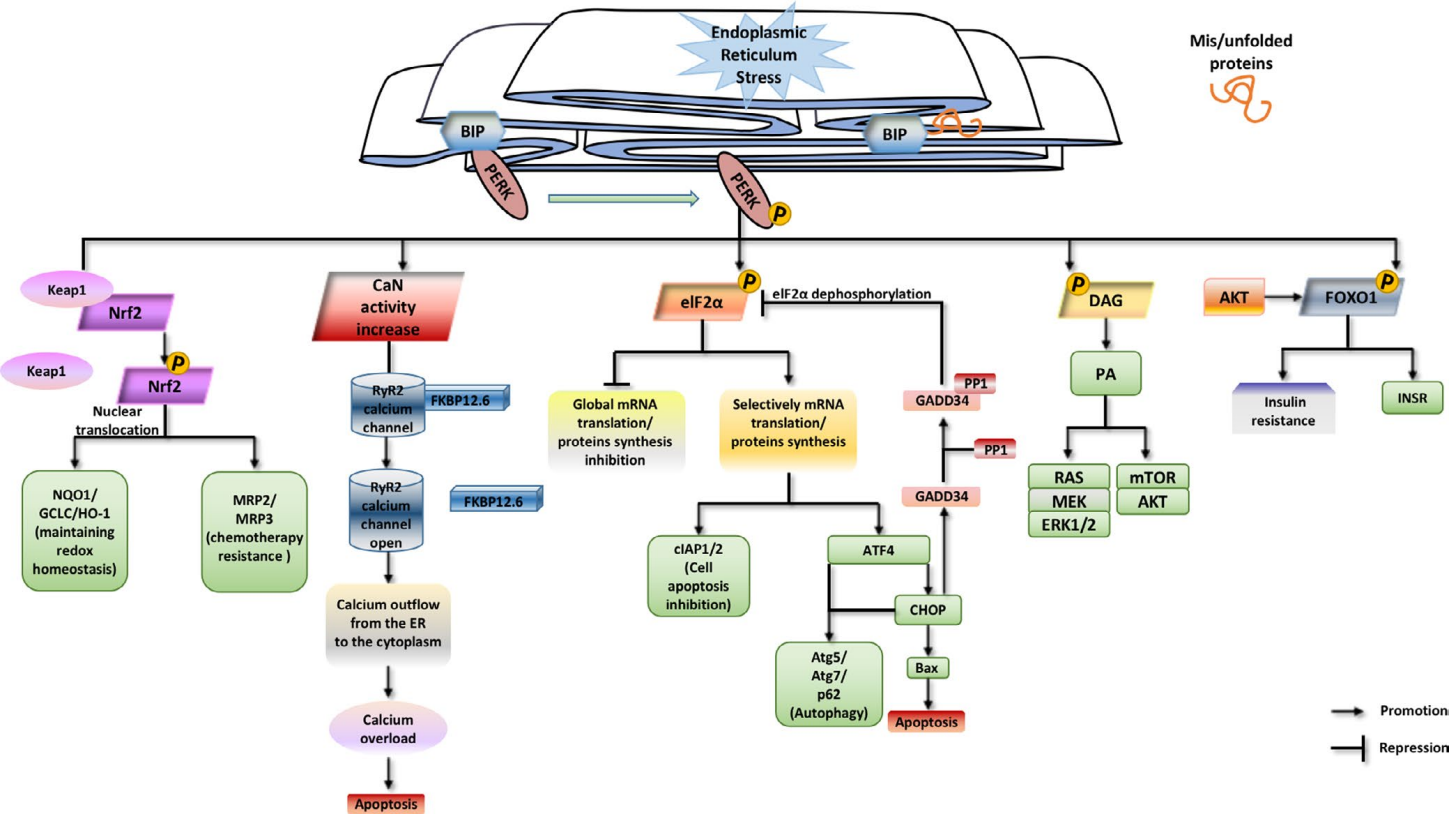
A brief diagram of the PERK signalling pathway. While ER stress is induced, unfolded protein competitively binds to BIP, causing BIP to dissociate from PERK and PERK is then phosphorylated. Phosphorylated PERK is activated and makes the downstream Nrf2 dislocate with Keap1 and activate, the activity of CaN increase, elF2α, DAG and FOXO1 phosphorylate, and they further play different roles respectively. Green squares indicate the expression of the genes

As a central regulator of ER stress, PERK decides cell fate by interacting with its own downstream molecules to form corresponding pathways (Figure 1).^26^ Under homeostatic conditions, PERK exists as an inactive monomer associated with binding immunoglobulin protein (BIP), which is also known as glucose‐regulated protein 78 (GRP78). Following exposure of cells to ER stress, BIP is released from PERK, thereby permitting PERK oligomerization and activation.^27^

### 2.1 PERK/eukaryotic initiation factor 2 (eIF2α)

One of the vital regulatory functions of PERK is its role as a monitor of protein translation, activation of PERK during ER stress will lead to a rapid decrease in the initiation of mRNA translation.^28^ The reduction of PERK‐dependent protein translation may limit the nascent protein transport to the ER lumen, reducing the potential molecular chaperone load and clearing the misfolding proteins. It is widely believed that this inhibitory effect is due to the phosphorylation of eIF2α at Ser51 by activated PERK, which disrupts the activation of 43S translation initiation complex formation, thereby reducing the rate of general protein translation.^29^, ^30^

### 2.2 PERK/nuclear respiratory factor 2 (Nrf2)

Nrf2 is a transcription factor of the CNC‐basic leucine zipper (CNC‐bZIP) family. The fact that cells lacking Nrf2 are extremely sensitive to compounds that induce ER stress, which highlights the importance of Nrf2 activation in response to ER stress.^31^ Sara et al pointed out that Nrf2 is one of PERK's direct substrate molecules. The PERK‐dependent phosphorylation of Nrf2 on threonine 80 can promote the dissociation of Nrf2 from Kelch‐like Ech‐associated protein 1 (Keap1), thereby translocating Nrf2 to the nucleus and activating the expression of its target genes.^32^ Importantly, this activation does not require the accumulation of reactive oxygen species (ROS) or the phosphorylation of eIF2α.^33^

### 2.3 PERK/calcineurin (CaN)

ER is considered to be a ‘calcium pool’ of cells, and under physiological conditions, the calcium channels associated with ER is inhibited. However, ER stress leads to depletion of ER calcium storage under pathological conditions.^34^ Previous studies reported that after inducing ER stress, autophosphorylated PERK increased the enzyme activity of CaN through direct interaction.^35^ Then, CaN promotes the dissociation of FKBP12.6 from the RyR2 calcium channel, triggering calcium outflow from the ER to the cytoplasm.^36^ The calcium overload regulated by the CaN/FKBP12.6 signalling can be considered as one of the possible mechanisms of ER stress‐induced apoptosis.^37^

### 2.4 PERK/forkhead box O 1 (FOXO1)

The modified FOXOs play an important role in regulating many cell processes including proliferation, differentiation, apoptosis and autophagy.^38^ Zhang et al have found that obesity‐induced ER stress acts on FOXO1 through PERK to enhance obesity‐induced insulin resistance, PERK can directly enhance FOXO1 activity to increase INSR levels and increase AKT activity.^39^, ^40^

### 2.5 PERK/diacylglycerol (DAG)

It is also known that PERK has lipid kinase activity and can phosphorylate lipid DAG, and DAG is the precursor of phosphatidic acid (PA) which produces a central node lipid second messenger. Previous study indicates that PERK‐dependent generation of PA is necessary to trigger signal transduction downstream of Ras in cells undergoing a strong ER stress response.^41^

## 3 PERK/eIF2α

### 3.1 PERK/eIF2α in osteoblasts

In the human body, mutations of the eukaryotic translation initiation factor 2α kinase 3 (EIF2AK3) gene (encoding PERK) can cause a rare autosomal recessive genetic disease: Wolcott‐Rallison syndrome (WRS), which features early‐onset type I diabetes, skeletal dysplasia and osteoporosis, this phenomenon indicates that PERK has a potential regulatory effect on the skeletal system.^42^ Global knocking out the *Perk* gene in mice is not embryonic lethal, but compared with wild‐type mice, *Perk* ^−/−^ mice at birth showed a marked reduction in the bone density of cortical bone and trabecular bone, and the second ossification centre of the proximal tibia is stunted.^43^ Wei et al speculated that osteopenia of global *Perk* ^−/−^ mice is caused by impaired osteoblast differentiation, insufficient number of mature osteoblasts and reduced type I collagen secretion. The expression levels of mature osteoblasts markers osteocalcin (OCN) and bone sialoprotein (BSP) were significantly reduced in *Perk* ^−/−^ osteoblasts. In addition, the collagen secretion level and alkaline phosphatase (ALP) activity of *Perk* ^−/−^ osteoblasts decreased, which shows that PERK has a significant influence on the late maturation of osteoblasts.^42^

Orthodontic tooth movement is the process of osteogenic differentiation of periodontal ligament stem cells (PDLSCs) and alveolar bone remodelling under the action of mechanical force.^44^, ^45^ Studies have shown that using Flexcell^®^ FX‐5000™ Tension System on PDLSCs, applying periodic mechanical stress under 10% deformation and 0.5 Hz (30 cycles/min) can induce the activation of PERK‐eIF2α‐ATF4 pathway and then upregulate the expression of BSP and OCN, thereby promoting the osteogenic differentiation of PDLSCs. However, the periodic mechanical stress had no obvious effect on bone formation of *Perk*‐knocked‐out PDLSCs.^46^ This reveals that the PERK‐eIF2α‐ATF4 signalling pathway mediated by ER stress is involved in the osteogenic differentiation of PDLSCs induced by periodic mechanical stress.

ATF4 is a transcription factor belonging to the cyclic adenosine monophosphate response element binding protein family and plays an important role in osteoblast differentiation.^47^ Saito et al have demonstrated that bone morphogenetic protein 2 (BMP2), which is necessary for osteoblast differentiation, can induce eIF2α phosphorylation in normal osteoblasts, resulting in increased ATF4 protein expression. However, BMP2 (100 ng/mL) failed to activate eIF2α phosphorylation and ATF4 expression in *Perk* ^−/−^ osteoblasts. Furthermore, in osteoblasts that specifically knocked out *Perk*, the decrease in ATF4 levels was accompanied by impaired activation of the specific cis‐acting element OSE1 site in the Ocn promoter.^48^ This result displays that PERK signalling is required for ATF4 activation during osteoblast differentiation. By overexpressing ATF4 in osteoblasts specifically knocked out of Perk, the phenomenon of bone mass reduction was almost completely reversed. In short, the PERK‐eIF2α‐ATF4 signalling pathway is activated during osteoblast differentiation, and ATF4, which is up‐regulated in the downstream of PERK, promotes OSE1 site‐dependent gene expression and then promotes osteogenesis.

Whereas, there is still some controversy about whether ATF4 is involved in the bone phenotype of *Perk* ^−/−^ mice. Saito et al^48^ found that the PERK‐eIF2a‐ATF4 signalling pathway was indeed activated in BMP2‐treated osteoblasts, and the expression of ATF4 was significantly reduced in osteoblasts that specifically knocked out *Perk*. However, Wei et al^42^ analysed the expression of ATF4 in skull osteoblasts collected from wild‐type and Perk^−/−^ mice, and no difference in ATF4 expression was observed between them. The reason for these apparent divergences is not clear yet, and other studies are still needed to clarify the specific regulation affection of PERK‐eIF2α‐ATF4 in osteoblasts.

### 3.2 PERK/eIF2α in osteoclasts

Mature osteoclasts release acid ions to absorb mineralized bone matrix and secrete proteases to degrade bone matrix proteins.^10^ Because the increase in the quantity of these enzymes may potentially stimulate ER stress, it was initially speculated that UPR was activated late in osteoclast differentiation.^25^ Nevertheless, some research results are contrary to this hypothesis. UPR is briefly induced during osteoclast differentiation and decays with the formation of osteoclasts.^49^ Treatment of RANKL‐induced BMMs with the ER stress activator thapsigargin can significantly promote the formation of osteoclasts, while silencing PERK suppresses this effect.^50^ The activity of osteoclasts in Perk knockout mice has been damaged, and the expression levels of Tartrate resistant acid phosphatase (TRAP) and Cathepsin K (CTSK) in the serum and bone extracts of Perk knockout mice are reduced compared with wild‐type mice.^42^ These studies forecast that ER stress occurs during osteoclastogenesis and PERK is involved in the regulation of osteoclast differentiation and activity.

eIF2α is involved in regulating osteoclast differentiation. Salubrinal is a compound, selectively inhibits protein phosphatase, which inhibits growth arrest and DNA damage‐inducing proteins. Salubrinal can block the dephosphorylation of eIF2α downstream of PERK.^51^, ^52^ Research by Li et al^53^ showed that Salubrinal (5‐20 μmol/L) inhibited the expression of Nuclear factor of activated T cells, cytoplasmic 1 (NFATc1) during the RANKL‐induced osteoclast differentiation of RAW264.7 cells; live cell imaging of RAW264.7 cells exhibited that Salubrinal (10 and 20 μmol/L) inhibited eIF2α‐induced Rac1 GTPase glycosylation and its activity, involving various functions including cell migration. In disuse osteoporosis animal model, Salubrinal has a significant inhibitory effect on the migration and adhesion of osteoclasts.^54^

According to the UPR signalling, promoting the phosphorylation of eIF2α will lead to enhanced ATF4 activity, ATF4 is an indispensable key molecule in osteoclast differentiation process.^55^ There also reports that the activation of the PERK‐eIF2α signalling has an acceleration effect on the expression of NFATc1 and osteoclastogenesis.^56^ Current studies found that eIF2α kinase contains four distinct serine‐threonine kinase families, which are PERK (EIF2AK3), PKR (EIF2AK2), general control non‐derepressible‐2 (GCN2, EIF2AK4) and haem‐regulated inhibitor (HRI, EIF2AK1), they respectively sense a variety of different cell stress responses, including viral infections, protein toxicity and low levels of essential nutrients (such as amino acids and haem).^57^ We speculate that the pharmacological phosphorylation impact of eIF2α dephosphatase inhibitor salubrinal actually is to activate a variety of stress signals in the cell, which does not accurately reflect the regulation effect of PERK‐eIF2α pathway during osteoclast differentiation. Therefore, we believe that further research is needed to clarify the expression change of eIF2α and the regulatory role of UPR mediated by PERK signalling during osteoclast differentiation.

## 4 PERK/Nrf2

### 4.1 PERK/Nrf2 in osteoblasts

Researches have exhibited that stable overexpression of Nrf2 in MC3T3‐E1 cells reduces the ALP activity and the mineralization level, and the recruitment of Runt‐related transcription factor 2 (RUNX2) on the osteocalcin promoter and RUNX2‐dependent osteocalcin promoter activity are also decreased.^58^, ^59^ This phenomenon is believed to be caused by Nrf2 interacting with RUNX2, inhibiting the binding of RUNX2 to OSE2 on the osteocalcin promoter and Nrf2 directly binding to ARE‐like‐2 element next to osteocalcin promoter blocking the transcription of osteocalcin. Besides, there are ARE‐like sequences in the mouse type I collagen promoter, hence it is speculated that type I collagen, which is the downstream of RUNX2, can also be negatively regulated by Nrf2 in these ways.^58^, ^59^ Nevertheless, some other studies have stated that the number of osteoblasts in *Nrf2* ^−/−^ mice is reduced.^60^ Similar results have been reached in the in vitro experiments, lack of Nrf2 weakened the viability of osteoblasts, and it was also found that knocking out Nrf2 in bone marrow stromal cells markedly increased the intracellular ROS.^61^ It is speculated that Nrf2 maintains bone homeostasis by assisting cells relieve oxidative stress. Rana et al^62^ proved that oxidative stress persisted in *Nrf2* ^−/−^ mice and the ability of osteoblasts to survive and differentiate was weakened, while the use of antioxidant *N*‐acetyl‐l‐cysteine (NAC) could alleviate this situation. It indicates that in the case where Nrf2 is reduced or deleted, the viability and differentiation of osteoblasts are influenced by the weakened ability of anti‐oxidative stress.

The above two types of opinions on the role of Nrf2 in osteoblast differentiation and activity seem to be divided, in fact, the disagreement can be explained, because a variety of osteogenic differentiation factors are in the optimal expression range to exercise the best pro‐osteogenic differentiation function, and the optimal expression range of cytokines is often narrow. Overexpression and complete knockout of *Nrf2* are not conducive to osteogenic differentiation, and the specific boundary expression level of Nrf2 on osteogenic differentiation needs further study to define.

### 4.2 PERK/Nrf2 in osteoclasts

Compared with the different views caused by the role of Nrf2 in osteoblastogenesis, the researchers have a unified view on the role of Nrf2 in osteoclast differentiation and activity.^60^ Treatment of osteoclast precursor cells overexpressing Nrf2 with RANKL shows inhibited osteoclast differentiation. Hyeon et al verified that *Nrf2* ^−/−^ BMMshad dysregulated ROS levels, and under the induction of RANKL, the formed osteoclasts were larger in volume than the wild type; they also found that dysregulation of anti‐oxidative stress and significant increase of ROS stimulated osteoclast activity. The increased ROS activates JNK, P38 and ERK1/2 in osteoclast precursors to promote osteoclastogenesis.^63^ Uniform with these, Sun et al^64^ demonstrated that the knockout of Nrf2 caused the increase of the osteoclast number in the distal femur of the mice and accelerated bone resorption. Moreover, there is a study also found that the lack of Nrf2 could cause increased expression of RANKL in osteoblasts.^65^ In addition, Keap1 deficiency indirectly leads to increased nuclear translocation of Nrf2, which will cause the expression of RANKL and the formation of osteoclasts to be blocked.^66^

In conclusion, the direct activation and nuclear translocation of Nrf2 can promote the antioxidant capacity of cell, which in turn inhibits osteoclast differentiation and activity. Similarly, suppressing keap1 to increase Nrf2 nuclear translocation can also have the same effect. Knockout or inhibition of Nrf2 will lead to weakened anti‐oxidative stress ability of cells, increased ROS level, promote osteoclast differentiation and activity.

## 5 PERK/CaN

### 5.1 PERK/CaN in osteoblasts

CaN is a Ca^2+^‐ and calmodulin‐activated serine‐threonine protein phosphatase that exists as a heterodimeric protein complex consisting of two subunits, the 61‐kDa calmodulin binding, catalytic subunit A and the 19‐kDa Ca^2+^ binding, regulatory subunit B. Three mammalian isoforms of calcineurin A (α, β and γ) and two B isoforms (1 and 2) have been identified. All CaN isoforms are expressed in osteoblasts.^67^

Overexpression of calcineurin Aα can promote osteoblast differentiation by increasing the expression of osteoblastogenesis markers Runx2, ALP, BSP and osteocalcin, while the loss or inactivation of CaN inhibits osteoblast differentiation.^67^ *Calcineurin Aα* ^−/−^ mice showed a marked reduction in bone formation rate and severe osteoporosis.^68^ The two commonly used immunosuppressants, cyclosporin A and tacrolimus (targeted binding to FKBP12), effectively inhibit the phosphatase activity of CaN by interacting with different domains of calcineurin A subunit. The systemic administration of either drug to rats causes severe osteoporosis by reducing osteoblast differentiation and bone formation.^69^ Without stimulation, NFAT remains in the cytoplasm in a highly phosphorylated state, and activation of CaN causes dephosphorylation of NFAT, leading to allosteric switch, which exposes the nuclear localization sequence and hides the nuclear export sequence. Then, NFAT translocates to the nucleus and binds to specific regions in the target gene promoters. NFAT signalling will coordinately regulate the expression of Wnt4, Frizzled9 and DKK2, thereby promoting the proliferation of osteoblasts.^70^, ^71^ However, recent studies suggest that the CaN‐NFAT pathway is a negative regulator of osteoblast differentiation and bone formation. The increase in bone mass was seen in mice lacking calcineurin B1 in osteoblasts. The loss of calcineurin B1 in primary osteoblasts significantly increased the expression of osteocalcin and reduced the dephosphorylation of NFATc1, which ultimately increased osteoblast differentiation.^72^ In addition, the loss of calcineurin B1 in osteoblasts increased the expression of OPG and decreased the expression of RANKL.^73^ It was found that low‐dose CaN antagonist cyclosporin A (<1 μmol/L in vitro and 35.5 nmol/L in vivo) increases osteoblast differentiation and bone mass, thus supporting the negative effect of CaN on bone formation.^74^ It is not fully understood why cyclosporin A treatment causes the opposite effects, but the difference in these results may be due to the use of different species, age, gender and, most importantly, the use of different drug concentrations in these studies and administration time. To be sure, research on the role of CaN in osteoblast proliferation and differentiation is limited.

### 5.2 PERK/CaN in osteoclasts

NFAT is a master transcription factor for osteoclast differentiation, functioning as a point of convergence for the TNF receptor‐associated factor 6 (TRAF6), RANKL and c‐Fos pathways required for osteoclastogenesis.^75^ Studies have shown that RANKL induces Ca^2+^ oscillation during osteoclast differentiation, leading to Ca^2+^/CaN‐dependent dephosphorylation and activation of NFATc1, which regulates serine residues in NFAT dephosphorylation leads to the exposure of nuclear localization signals and subsequent transfer to the nucleus to induce osteoclast‐specific gene transcription, thereby causing osteoclast differentiation.^76^, ^77^ Long‐term Ca^2+^ oscillations are thought to maintain NFATc1 expression in the nucleus and ensure long‐term transcriptional activation of NFATc1 required for terminal differentiation during osteoclast formation. In short, Ca^2+^ oscillation is a key feature of signalling in osteoclastogenesis.

CaN plays a key role in regulating the occurrence of osteoclastogenesis, but current research has found that its role in osteoclast formation and function seems to be different. CaN can positively regulate osteoclast formation, but CaN activation in mature osteoclasts tends to reduce resorption activity. The mechanism may be through the feedback effect of extracellular Ca^2+^ to inhibit excessive bone resorption.^68^

## 6 PERK/FOXO1

### 6.1 PERK/FOXO1 in osteoblasts

The research of Teixeira et al Showed that FOXO1 is a positive regulator in the early process of osteogenic differentiation of mesenchymal cells. Osteogenic induction factor BMP2 (100 ng/mL) enhances the activity and expression of FOXO1 in mouse embryonic mesenchymal cells (C3H10T1/2 cells), and overexpression of FOXO1 will significantly increase the expression level of osteogenic markers, such as Runx2, Alp and osteocalcin (Ocn). Conversely, silencing FOXO1 inhibits the expression of these osteogenic markers, impairs bone formation and especially reduces bone development in the craniofacial region.^78^ In addition, studies have shown that FOXO1 can interact with ATF4, and this interaction enhances the transcriptional activity of FOXO1 and ATF4.^79^, ^80^

In the above example, FOXO1 regulates bone formation by enhancing osteoblast differentiation and maintaining cell health by inhibiting oxidative stress. However, in other cases, FOXO1 may negatively affect bone formation by affecting Wnt signalling. Previous study found that binding of β‐catenin to FOXOs diverts the limited pool of β‐catenin from Wnt/TCF to FOXO‐mediated transcription and decreases osteoblastogenesis in vitro. Knockout of FOXO1 in the progenitors of osteoblast will increase osteoblast differentiation.^81^, ^82^ Some scholars believe that this phenomenon is due to enhanced cell proliferation and reduced FOXO1 interference with Wnt/β‐catenin signalling. In addition, FOXO1 may contribute to the immune‐mediated inhibition of bone formation by promoting the apoptosis of osteoblasts.^83^ In summary, the effect of FOXO1 on osteoblast is complex and unclear.

### 6.2 PERK/FOXO1 in osteoclasts

Specific deletion of FOXO1 in BMMs reduces M‐CSF‐induced RANK expression and migration of BMMs and the loss of FOXO1 in osteoclast precursors will reduce the expression and nuclear localization of NFATc1.^84^ Past studies have shown that RANKL can increase the level of ROS in BMMs, and ROS can enhance osteoclast differentiation.^85^ However, the mechanism that causes increased ROS to promote osteoclast differentiation remains unclear. Studies using a mouse model of loss FOXOs in osteoclasts have clarified that FOXOs is an important negative regulator of osteoclast differentiation and function by reducing ROS. In fact, the combined loss of FOXO1, 3 and 4 in osteoclast progenitors increases proliferation, osteoclast formation and bone resorption, resulting in a reduction in trabecular and cortical bone mass. RANKL reduces the level and activity of FOXO1, 3 and 4 through Akt‐mediated phosphorylation and proteaosomal degradation, thereby accumulating ROS levels.^86^ FOXOs also stimulates the expression of blood haem oxygenase 1 (HO‐1) in osteoclast precursors.^87^

The above studies have produced conflicting results in clarifying the role of FOXO in osteoclastogenesis. Tan et al set out to resolve these discrepancies and better understand the role of FOXO1 in osteoclast differentiation and function. In their study, they confirmed that FOXO1 is an inhibitor of osteoclast differentiation and function, and provided new insights into the mechanism. They found that FOXO1 expression was reduced after RANKL treatment and FOXO1 inhibition promoted osteoclast differentiation and activity.^84^ By showing that pretreatment with ROS scavengers NAC or MYC inhibitors 10058‐F4 abolished the inhibitory effect of FOXO1, study have demonstrated that the inhibitory effect of FOXO1 on osteoclast differentiation and function is partially mediated by inhibition of ROS generation and MYC.

## 7 PERK/DAG

### 7.1 PERK/DAG in osteoblasts

Studies have shown that the continuous increase in the production of 1, 2‐DAG induced by prostaglandin F2A (PGF2A) is important for the proliferation of osteoblast‐like MC3T3‐E1 cells. It is considered that the activation of PERK can promote the generation of PA by DAG, thereby reducing the accumulation of DAG in cells.^88^ Therefore, the activation of the PERK/DAG pathway may promote the proliferation of osteoblasts, but there is not enough research data to show that the PERK/DAG pathway affects the proliferation and differentiation of osteoblasts.

### 7.2 PERK/DAG in osteoclasts

DAG is a crucial second messenger involved in a variety of cellular reactions including secretion, motility, differentiation and proliferation.^89^ Evidence suggests that DAG plays a vital role in regulatingosteoclast differentiation. The main function of the PLCγ family is to cleave phosphatidylinositol 4,5‐bisphosphate (PIP2) into two secondary messengers, inositol 1,4,5‐trisphosphate (IP3) and DAG. Genetic deletion of PLCγ2 leads to bone loss due to inhibition of osteoclast differentiation.^90^ Ablation of DAG‐dependent protein kinase C delta (PKCδ) leads to defective bone resorption and high bone mass.^91^ All these findings indirectly indicate that DAG is a vital regulator of osteoclastogenesis.

Diacylglycerol kinase ζ (DGKζ) strictly regulates the level of DAG in the osteoclast precursor by converting DAG to phosphatidic acid, so lack of DGKζ will lead to DAG accumulation. Studies have shown that accumulation of DAG through DGKζ deletion can lead to a substantial osteoporotic bone phenotype. Surprisingly, increased DAG contributes to upregulation of the osteoclastogenic transcription factor c‐Fos in response to M‐CSF but not RANKL. The c‐Fos promoter region contains four enhancer elements, and studies have shown that the TRE site activates c‐Fos transcription in response to DAG analogs.^92^ PERK and DGKζ have similar functions and can convert DAG to phosphatidic acid.^41^ We speculate that activated PERK promotes the phosphorylation of DAG to form phosphatidic acid, which can reduce the accumulation of DAG in BMMs, which may inhibit the formation of osteoclasts.

## 8 PERK PATHWAY‐RELATED BONE METABOLIC DISEASES

### 8.1 Wolcott‐Rallison syndrome

Wolcott‐Rallison syndrome is a rare autosomal recessive genetic disease caused by mutations in the gene encoding EIF2AK3, which is characterized by abnormal bone development, growth retardation and neonatal/early‐onset non‐autoimmune insulin‐demanding diabetes.^93^ Scientists now generally believe that the marriage of close relatives is the most common cause of WRS.^43^

Approximately 30%‐40% of the *Perk* ^−/−^ mice died prenatally. Surviving newborn *Perk* ^−/−^ mice are normal in size, but show a remarkably similar phenotype to human WRS, including growth retardation, permanent neonatal diabetes, hepatic dysfunction, skeletal dysplasia and exocrine pancreas deficiency. PERK is highly expressed in bone tissue at levels comparable to those of the pancreas, which indicates that PERK has a potential developmental or physiological role in the skeletal system. *Perk* ^−/−^ mice exhibit severe spine flexion (hunchback), hindlimb splay and decreased exercise capacity.^42^ Therefore, PERK is indispensable in bone tissues, which are organs that have high requirements for protein synthesis and processing, and are susceptible to high and/or long‐term ER stress and ageing. This conclusion provides strong evidence that loss of PERK can lead to the phenotype of WRS, a complex disease.

*Perk* ^−/−^ mice showed a decrease in type I collagen α1 and α2 (COLIA1 and COLIA2), but the corresponding type I collagen precursors in *Perk* ^−/−^ bone tissue accumulated at abnormally high levels, indicating the deficiency in the mature type I collagen is due to the inhibition of procollagen processing or transport of the ER/Golgi secretory pathway. In addition, fragmented and swelled ER‐containing electron‐dense substances appeared in osteoblasts.^42^ The researchers speculate that in the case of PERK knockout, the secretory pathway in osteoblasts is damaged, and collagen cannot be efficiently processed and secreted, resulting in the accumulation of collagen and the expansion of the ER pool. Patients with osteogenesis imperfecta may be associated with reduced osteoblast collagen synthesis.^94^

On the basis of the abnormal general bone structure of *Perk* ^−/−^ mice, the researchers found defects in the formation of dense bones of the long and flat bone types. The compact bone of the long bone collar, the body of the vertebrae and the parietal bone of the skull show large perforations and discontinuities, impairing their structural integrity. These perforations and discontinuities allow the bone marrow to escape from the medullary cavity. The epiphyseal growth plate of *Perk* ^−/−^ mice seems normal. However, in the absence of normal compact bone, spongy trabecular bone often exhibits compensatory growth to provide structural integrity. Most bone defects in *Perk* ^−/−^ mice occur in the compact bone matrix secreted by osteoblasts. Ultrastructural examination revealed that many (but not all) *Perk* ^−/−^ osteoblasts showed highly expanded and fragmented ER, and some osteoblasts showed signs of apoptosis.^43^

Given the ER dysfunction observed in WRS patients, intervention strategies aimed at reducing ER stress or other pathways involved in this dysfunction may have potential effects for the treatment and prevention of WRS.

### 8.2 Rheumatoid arthritis

Rheumatoid arthritis (RA) is an autoimmune‐related chronic inflammatory disease characterized by chronic inflammation of synovial lining cell hyperplasia, which destroys bone by promoting the activation of osteoclasts.^95^ Some studies have shown that the accumulation of unfolded protein in the ER is detected in the synovial cells of RA.^96^, ^97^ Therefore, ER stress may be closely related to the pathogenesis of RA. In the synovial tissues and macrophages of RA patients, increased expression levels of the *EIF2AK3* gene (encoding PERK protein) and phosphorylated eIF2α were observed.^98^ Recent study reported that, in peripheral blood mononuclear cells of RA patients, the expression level of GADD34 (the downstream target of PERK/eIF2α/ATF4 pathway) has increased, and it is related to the production of proinflammatory cytokines.^99^

Salubrinal is an indirect blocker of eIF2α dephosphorylation, which can protect cells from ER stress‐induced apoptosis by increasing the level of phosphorylated eIF2α, thereby further inhibiting protein synthesis.^52^ The researchers found that salubrinal (5‐10 μmol/L) inhibited the pathological progress of RA by inhibiting the dual specific phosphatase (Dusp 2), a phosphatase that phosphorylates mitogen‐activated protein kinase. In addition, the administration of Salubrinal (2 mg/kg) can not only block inflammatory cytokines in immune cells, such as IL‐2, IL‐13, IL‐1β and TNF, but also alleviate CIA‐induced inflammation in mouse joints.^100^

### 8.3 Osteoporosis

Osteoporosis (OP) is a systemic skeletal disease that is common in the middle‐aged and elderly population. It is characterized by damage to the microstructure of bone tissue and a reduction in bone mass, which in turn leads to an increased risk of fracture.^101^ As a worldwide epidemic, according to statistics, there are more than 200 million OP patients worldwide.^102^

ER stress plays a key role in the pathogenesis of osteoporosis, and salubrinal has a good therapeutic effect on osteoporosis induced by different incentives. Electron microscopy showed that ovariectomy induced an increase in rough ER and a decrease in ribosome population on the ER membrane in osteoblasts and salubrinal (1mg/kg) can inhibit the expansion of rough ER induced by ovariectomy.^53^ In the model of glucocorticoid (GC)‐induced bone loss, part of the pro‐apoptotic effect of GC on osteoblasts is mediated by ER stress, and GC significantly reduces mineralization in OB‐6 cells or primary osteoblasts; salalbrinal (1‐100 μmol/L) or guanabenz (10 μmol/L) (specific inhibitor of eIF2α dephosphorylation) increases mineralization and prevents the inhibitory effect of GC.^103^ In short, salalbrinal prevents GC‐induced osteoblast apoptosis in vitro and in vivo and the harmful effects of GC on the skeleton. In disuse osteoporosis, ER stress caused by unloading is significantly inhibited by salalbrinal. By inhibiting the dephosphorylation of eIF2α, the use of salubrinal (1‐5 μmol/L) can effectively reduce apoptosis, as well as stimulate osteoblast differentiation and inhibit osteoclast differentiation.^54^

Parathyroid hormone (PTH 1‐34) is one of the anabolic osteoporosis drugs approved by the Food and Drug Administration in the United States. PTH (500 ng/mL) induces ER stress and promotes the expression of ATF4 by activating ER stress‐related PERK‐eIF2α signalling in osteoblasts. In view of the core role of ATF4 protein in the regulation of key genes for bone formation, activation of PERK‐EIF2α‐ATF4 signalling may help PTH regulate osteoblast differentiation and proliferation. Using one verified siRNA for PERK and two different inhibitors (ISRIB and AMG’44), the resreachers demonstrated that PERK plays a pivotal role in PTH‐mediated osteoblast differentiation and proliferation. Since it has been reported that salubrinal regulates the differentiation of osteoclasts and osteoblasts in osteoporosis by inhibiting the dephosphorylation of eIF2α, so it was not surprising to find that salubrinal can enhance the PTH‐induced osteoblast differentiation and proliferation.^104^ Although phosphorylation of EIF2α inhibits global protein translation, it also specifically activates ATF4 translation. It seems that the enhancement effect of salubrinal is largely dependent on the expression of ATF4, because knockout of ATF4 diminishes salubrinal‐induced osteoblast differentiation.^105^ Suppression of global translation by salubrinal reduces the loading of newly synthesized proteins into the ER, which may contribute to the improvement of protein‐folding in the ER. However, this mechanism needs further study to verify this possibility.

Regulation of osteoclast differentiation and function can prevent osteoporosis under inflammatory conditions. Wang et al demonstrated that ER stress is related to the differentiation of osteoclast precursor cells. Thapsigargin (TG) is an ER stress inducer that promotes the formation of osteoclasts in a dose‐dependent manner (0.05‐0.5 nmol/L), depending on crosstalk with the RANKL signalling pathway, including NF‐κB activity and ROS production. But silencing PERK can reverse the effect of TG on promoting osteoclast differentiation.^56^

### 8.4 Osteonecrosis of the femoral head

Osteonecrosis of the femoral head (ONFH) is a serious orthopaedic disease related to the hip joint. The cause of femoral head osteonecrosis due to impaired blood supply to the femoral head, and then, the femoral head collapses.^106^ Risk factors for inducing this disease include trauma, excessive alcohol intake, Legg‐Calve‐Perthes disease and use of corticosteroids. Glucocorticoids (GCs) are one of the most common causes of non‐traumatic ONFH.^107^ According to reports, osteonecrosis occurs in 9%‐40% of patients receiving long‐term GC therapy.^108^ Due to the lack of effective drugs for ONFH, patients with late stage of this disease often need to undergo hip replacement surgery. The long duration of this disease brings continuous pain and financial burden to patients.

ER stress plays a unique role in ONFH and may act as a two‐edged sword. Although moderate levels of ER stress may contribute to the restoration of cellular homeostasis, the long‐term and unmitigated ER stress may lead to cell apoptosis.^25^ In the current model of ONFH, the ischaemic femoral head cannot obtain enough blood, nutrients or oxygen, and the excessive ER stress induced.^109^ Research results have shown that osteonecrosis increases the expression of p‐eIF2α, and the increase in ATF4 levels caused by ER stress can promote osteoclast differentiation. In addition, osteonecrosis activates osteoclast differentiation, migration, adhesion and activity. The degree of blood perfusion is negatively correlated with osteoclastogenesis.^110^ Overall, ischaemia in ONFH induces osteoclast differentiation and interrupts bone healing by inducing sustained and high‐strength ER stress.

Sato et al^103^ have reported that when GC or ER stress inducers induce apoptosis, salubrinal or guanabenz can block apoptosis of osteocytic MLO‐Y4 and osteoblastic OB‐6 cells. Liu et al^110^ reported that ER stress is an important pathological result of ONFH animal modelling, and salubrinal inhibits osteoclastogenesis by reducing the level of NFATc1 as well as promotes the activity of osteoblasts by increasing the level of ATF4. The above two studies have shown that inhibition of eIF2α phosphorylation can reduce ER stress and has been shown to effectively slow the pathological progress of ONFH.

## 9 CONCLUSION AND PROSPECTS

**FIGURE 2:**
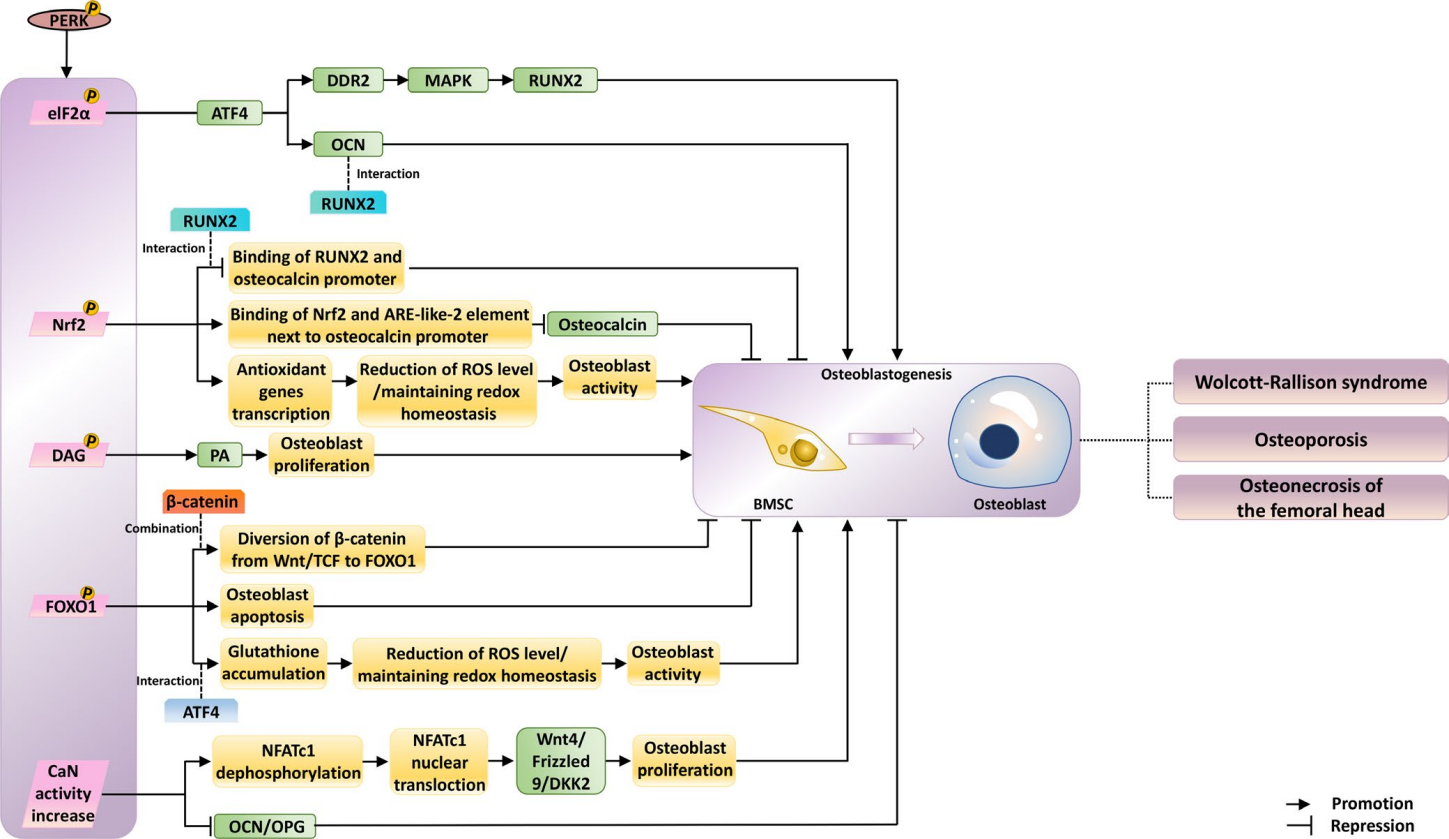
Effects of downstream changes caused by activation of PERK on osteoblastogenesis. Phosphorylated elF2α, Nrf2, DAG, FOXO1 and enhanced activity of CaN have different impacts on osteogenic differentiation, respectively. Highly related diseases are also showed. Green squares indicate the expression of the genes

**FIGURE 3:**
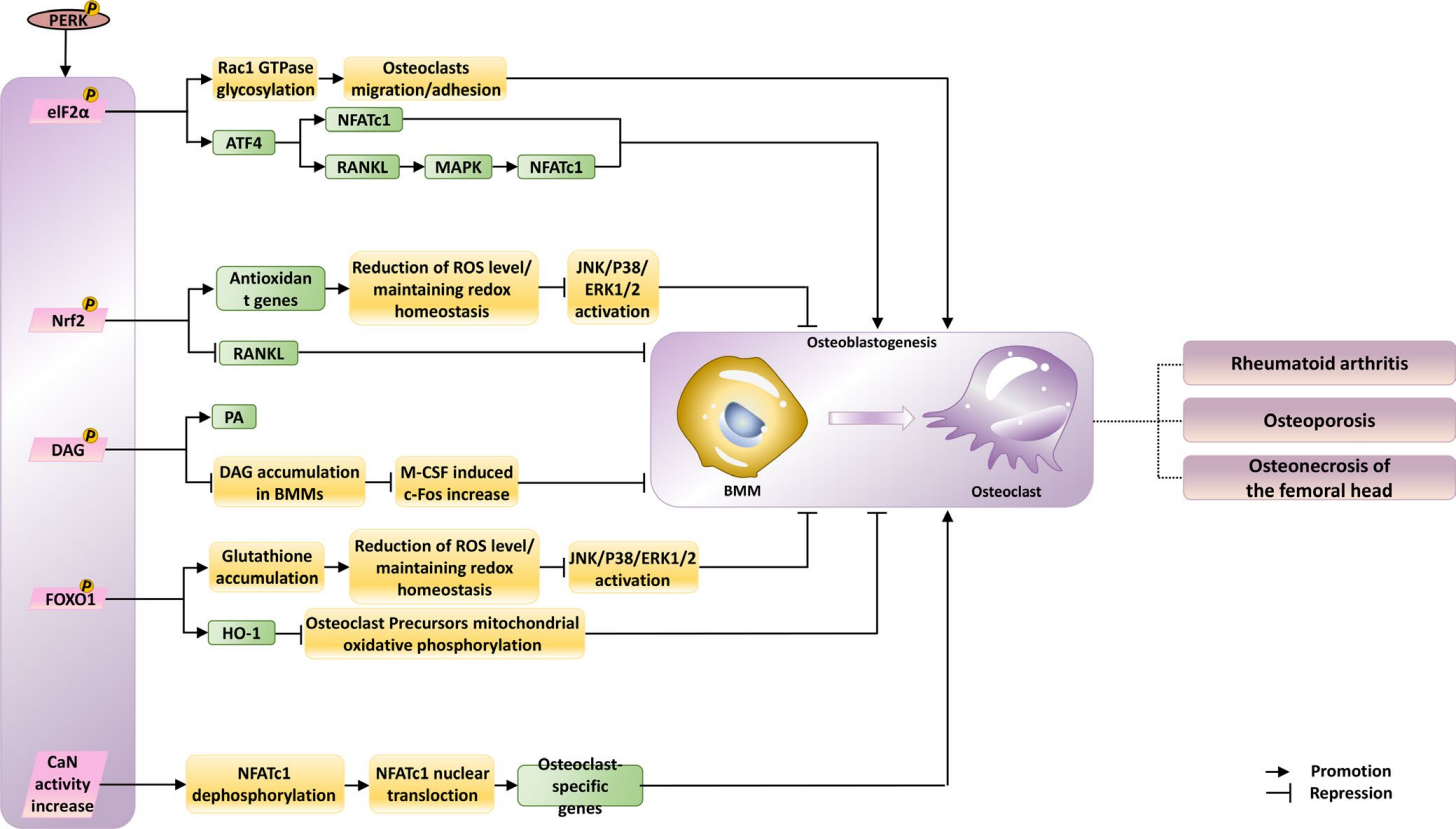
Impact of PERK signalling activation on osteoclastogenesis. PERK activation‐induced phosphorylated elF2α, Nrf2, DAG, FOXO1 and enhanced activity of CaN play different roles on osteoclast differentiation, respectively. Strongly associated diseases are exhibited. Green squares indicate the expression of the genes

In this review, we have described the current evidence that highlights the role of components of the PERK pathway in maintaining bone homeostasis. However, there is still a need for further research to develop new treatments, which will help find cure for bone diseases. Based on the current evidence, we summarized the main mechanisms involved in bone metabolism in the downstream of PERK pathway, among which DAG, FOXO1, CaN, Nrf2 and elF2α play a role in regulating the differentiation of osteoblasts and osteoclasts (Figures 2 and 3). In order to better develop drugs targeting these proteins, more functional experiments and in vivo experiments should be conducted under various pathological conditions. Fortunately, several compounds have been found to regulate the PERK pathway, directly or indirectly contributing to the attenuation of bone‐related diseases. It is worth noting that the safety and effectiveness of these drugs have not been guaranteed by clinical trials.

In summary, the PERK pathway is essential in bone metabolism under physiological and pathological conditions. Therefore, those important regulators and compounds that target the PERK pathway have a promising application in the research and clinical practice of a series of diseases. The development of research in this field will bring a bright future for the treatment of bone‐related diseases.

## CONFLICT OF INTEREST

The authors declare that they have no conflict of interest.

## AUTHOR CONTRIBUTIONS

Jiachao Guo and Ranyue Ren wrote the manuscript and provided the critical revisions. Ranyue Ren and Kai Sun collected the update reference and drew the figures. Jinpeng He and Jingfan Shao provided the conception and design of the study.

## Data Availability

All the data are available from the corresponding author by request.
